# Medical practices display power law behaviors similar to spoken languages

**DOI:** 10.1186/1472-6947-13-102

**Published:** 2013-09-04

**Authors:** Jonathan D Paladino, Philip S Crooke, Christopher R Brackney, A Murat Kaynar, John R Hotchkiss

**Affiliations:** 1 Department of Medicine, John A. Burns School of Medicine, Honolulu, HI, USA; 2 Department of Mathematics, Vanderbilt University, Nashville, TN, USA; 3 Veteran’s Affairs Healthcare System, Pittsburgh, PA, USA; 4 Department of Critical Care Medicine, University of Pittsburgh, Pittsburgh, PA, USA; 5 Veteran’s Engineering Resource Center, Pittsburgh, PA, USA

**Keywords:** Power law, Natural languages, Mechanical ventilation, Dialysis, Medical practices

## Abstract

**Background:**

Medical care commonly involves the apprehension of complex patterns of patient derangements to which the practitioner responds with patterns of interventions, as opposed to single therapeutic maneuvers. This complexity renders the objective assessment of practice patterns using conventional statistical approaches difficult.

**Methods:**

Combinatorial approaches drawn from symbolic dynamics are used to encode the observed patterns of patient derangement and associated practitioner response patterns as sequences of symbols. Concatenating each patient derangement symbol with the contemporaneous practitioner response symbol creates “words” encoding the simultaneous patient derangement and provider response patterns and yields an observed vocabulary with quantifiable statistical characteristics.

**Results:**

A fundamental observation in many natural languages is the existence of a power law relationship between the rank order of word usage and the absolute frequency with which particular words are uttered. We show that population level patterns of patient derangement: practitioner intervention word usage in two entirely unrelated domains of medical care display power law relationships similar to those of natural languages, and that–in one of these domains–power law behavior at the population level reflects power law behavior at the level of individual practitioners.

**Conclusions:**

Our results suggest that patterns of medical care can be approached using quantitative linguistic techniques, a finding that has implications for the assessment of expertise, machine learning identification of optimal practices, and construction of bedside decision support tools.

## Background

Medical care commonly involves the apprehension of complex patterns of patient derangements to which the practitioner responds with patterns of interventions, as opposed to single therapeutic maneuvers. This complexity renders the objective assessment of practice patterns using conventional statistical approaches difficult. Combinatorial approaches drawn from symbolic dynamics can be used to encode the observed patterns of patient derangement and associated practitioner response patterns as sequences of symbols [1-7]. Concatenating each patient derangement symbol with the contemporaneous practitioner response symbol creates “words” encoding the simultaneous patient derangement and provider response patterns and yields an observed vocabulary with quantifiable statistical characteristics [7].

We reasoned that, if patient physiologic derangements are encoded as symbol streams and the provider responses are similarly characterized, patterns of medical practice may be quantitatively analyzed using linguistic approaches such as counting the frequency with which particular “words” (integrated symbols) appear. We applied this approach to individuals (subjects) managing simulated patients undergoing mechanical ventilation, as well as to the practices of experienced providers managing anemia in the setting of End Stage Renal Disease, two entirely unrelated clinical settings characterized by vastly different time scales.

A fundamental observation in many natural languages is the existence of a power law relationship between the rank order of word usage and the absolute frequency. In particular, suppose that we have *N* distinct words and one counts the frequency of each word and forms a histogram of the frequencies of the words, starting from the most frequently used word to the word of least frequency. Let *f*_*i*_ denote the frequency of the ***i***^*th*^ word and order the frequencies so that *f*_1_ ≥ *f*_2_ ≥ _∙∙∙_ ≥ *f*_*N*_. With each frequency *f*_*i*_, associate a rank *r*_*i*_∈{1,2,_∙∙∙_,*N*}; hence, *r*_*i*_ = *i*. The frequencies satisfy *Zipf-like Law* if

$$f_{i}=\frac{\beta }{r_{i}^{a}},\;i=1,2,\ldots ,N$$

where *a* and *β* are positive constants. Alternatively, this relationship could be expressed as

$$\ln\left(f_{i}\right)=-a\ln\left(r_{i}\right)+\ln\left(\beta \right).$$

**Figure 1:**
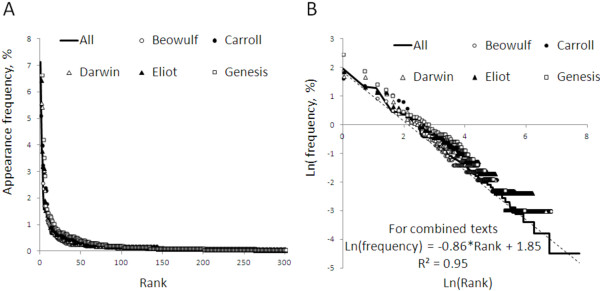
**Written languages display power law behavior in word frequency.** Panel **A**: word appearance frequency as a function of word use rank in Beowulf, Through the Looking Glass (Lewis Carroll), The Origin of the Species (Charles Darwin), The Love Song of J Alfred Prufrock (TS Eliot), Genesis (King James Version), and the combined lexicon. Panel **B**: natural logarithm (appearance frequency) versus natural logarithm (rank) for words in each text and the combined lexicon.

Plotting the variable *y* = ln(*f*) and *x* = ln(*r*) gives a linear relations between *x* and *y* slope − *a* and *y-*intercept at ln(*β*). Figure 1 contains illustrations for text taken from famous literary works [8-10].

Our work investigates the usage of “words” in 2 different domains of medical practice and the degree to which word usage displays Zipf-like laws. In the following sections we will discuss what constitute words for each domain and the extent to which their frequencies satisfy ln (*f*_*i*_) = *-a* ln (*r*_*i*_) + ln (*β*).

## Methods

The general algorithm for encoding patient derangements, provider responses, and integrated derangement response patterns as symbols is depicted in the subsection below that demonstrates the encoding steps. Analyses were performed using either *Mathematica* 9 (Wolfram Research, Champaign, IL, USA) or *Visual Basic for Applications* running in *Microsoft Excel* (Redmond, Washington, USA). The mechanical ventilation study was deemed anonymous Exempt Educational Research by the University of Pittsburgh and University of Hawaii Institutional Review Boards; the dialysis investigation was approved as Minimal Risk by the Veterans Affairs- Pittsburgh Institutional Review Board. For the textual examples, source files were obtained from the integrated *Mathematica* database.

### Word usage in works of literature

Text files for the first ~ 2000 words of Beowulf, Through the Looking Glass (Lewis Carroll), The Origin of the Species (Charles Darwin), and Genesis (King James Version), as well as the first ~ 1100 words of The Love Song of J Alfred Prufrock (TS Eliot) were obtained from the Wolfram Research database. These files were converted to tab delimited strings after elimination of punctuation marks. For each corpus, a dictionary of all observed words was constructed; these were combined to form a total lexicon. For each work, and for the total lexicon, the appearance frequency of each word was determined and these appearance frequencies were rank ordered to generate the figures.

### Mechanical ventilation

We applied symbolic dynamics approaches to analyze practice patterns germane to managing machine-based respiratory support in patients unable to breathe independently (mechanical ventilation). Mechanical ventilation is a lifesaving but potentially harmful intervention in which a practitioner adjusts machine support parameters (such as oxygen concentration, breath frequency, and breath size) on a minute-by-minute or day-by-day basis. Using previously developed models of mechanical ventilation, a digital training simulator was developed that confronted subjects with a physiologically realistic population of 100 dynamically active “virtual patients,” in concert with defined physiologic goals [11-17]. All sequential physiologic values taken on by the virtual patients, as well as the sequential adjustments made by the subjects, were recorded. A more recent version of the simulator can be downloaded, free of charge, from http://www.math.vanderbilt.edu/~pscrooke/CANVENT/upload.html.

Elements of the pattern of physiologic derangement comprised oxygen saturation (goal: > 90%), pH (goal: lower bound < pH <7.45, where lower bound is disease process specific), mean arterial blood pressure (goal: > 65 mmHg), and end-of-breath distending pressure in the airspaces (goal: < 30 cmH_2_O); subjects were provided goal ranges for each of these variables for each virtual patient. Elements of the intervention pattern comprised inspired concentration of oxygen, applied end expiratory pressure, breath frequency, and mode of ventilation (pressure controlled/time cycled or volume controlled). For pressure controlled ventilation, applied inspiratory pressure and inspiratory time were recorded; for volume controlled ventilation breath size, inspiratory gas flow rate, and inspiratory pause time were recorded.

Combinatorial techniques were applied to encode the pattern of patient derangement prior to each adjustment, as well as the integrated pattern of interventions implemented by the subject at that point. Contemporaneous symbols (disorder and intervention) were combined to define “words” encoding the disorder: intervention pattern. 29 subjects ranging from trainees to experienced faculty members each managed 100 virtual patients, attempting to attain pre-specified and explicit physiologic endpoints. The algorithm that is used to construct the derangement: practitioner intervention words is summarized in a word-algorithm (see Additional file 1).

### Dialysis

The second domain of medical interaction analyzed was the management of anemia in End Stage Renal Disease, an area of great clinical scrutiny with shifting clinical goals generally encompassing a hemoglobin value of greater than 10 and less than 12 g/L. Therapeutic adjustments–such as increasing the dose of erythropoiesis stimulating agents or the administration of iron–are made on a time scale of weeks to months.

Elements of the patient patterns for these analyses included hemoglobin and ferritin concentrations, iron saturation, tertile of erythopoiesis stimulating agent dosage, rate of change in hemoglobin from the previous month, and the difference between current hemoglobin and the specified target range. The target ranges used in the analysis were hemoglobin 10–12; iron saturation >20%; and ferritin > 200 and less than 500. Elements of the provider response pattern included changes in erythropoiesis stimulating agent (start, stop, increase or decrease by 25 to 50%, increase or decrease by >50%) and iron management (start, stop, or intensify).

A database interrogation tool was constructed to capture the prevailing pattern of derangement in each patient, as well as the pattern of response implemented by the practitioner in response to the observed ensemble of patient derangements. The database incorporated preexisting data from approximately 30 patients in a medium size metropolitan dialysis unit who were followed over a 3-year period. Management was implemented by nine trainees and one Advanced Practice Registered Nurse, all supervised by one of five senior attending physicians who rotated in through the unit on a monthly basis. There is not a specific anemia treatment protocol in this dialysis unit.

### Intermittent silence analysis of mechanical ventilation

We now address an interesting question that is based on earlier work by Miller: to what extent might the observed patterns reflect a statistical process driven by random selection of “keystrokes” (selected interventions) punctuated by intermittent spaces [18]? This is equivalent to a generating model in which “…a monkey hits the keys of a typewriter at random…” and the length distribution of the terms thus produced is conditioned by the number of keys and the probability of striking the space key. Miller’s argument was subsequently formalized by Li [19]. We addressed this question both for the individual interventions implemented and for the lengths of solutions observed for the virtual patients.

To investigate the patterns displayed in individual interventions during simulated volume controlled mechanical ventilation, we conceptualized a keyboard having 14 keys: 12 setting change keys (increase tidal volume, decrease tidal volume; increase frequency, decrease frequency, *etc*.), one key for a fluid bolus, and one space bar (no other interventions). For each parameter, only the presence of a change in a setting was considered, not the magnitude of the change itself. A similar approach was implemented for pressure controlled ventilation, albeit using the 10 setting changes possible in this mode, one key for a fluid bolus, and one space bar. The monkey randomly strikes keys on the keyboard until the space key is struck, and the number of inputs changed (up to 7 in volume controlled ventilation and 6 in pressure controlled ventilation) corresponds to the word length in Miller and Li’s work, and is denoted by “intervention pattern length.” The rank ordered distributions of intervention pattern lengths > 0 for volume cycled and pressure control ventilation were determined separately. These were compared to the distributions expected from Miller’s intermittent silence and Li’s random text analyses.

To address solution lengths, we considered the lengths of “solutions:” sequences of interventions required to complete the solution (or non-solution) of an individual virtual patient in the mechanical ventilation data. The number of interventions required to complete each virtual patient was counted, yielding a distribution of solution lengths that were then rank ordered by appearance frequency, and the effective frequency of a “space” was determined within the dataset. For the combination of volume controlled and pressure controlled ventilation there were 1946 discrete possible interventions (1458 in volume controlled + 486 in pressure controlled + 2 for changes between modes), corresponding to the “keys” in [18] and “symbols” in [19]. Only solutions of length greater than or equal to 1 and having a total appearance count >1 were considered. The values obtained were used to parameterize equations 6 of [18] and [19], and observed behaviors versus those anticipated from “intermittent silence” and “random text” processes were compared.

## Results and discussion

### Mechanical ventilation

**Figure 2:**
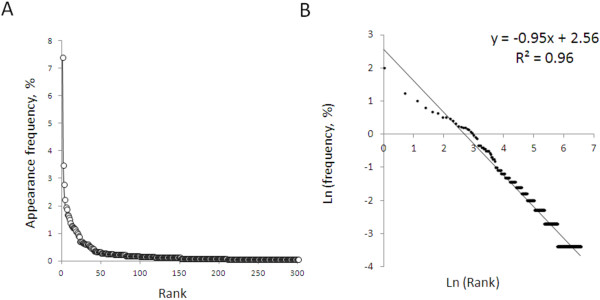
**Practice patterns during management of simulated patients with mechanical ventilation display language like statistics.** Panel **A**: word appearance frequency versus word appearance rank during simulated practice of mechanical ventilation. Panel **B**: natural logarithm (frequency) versus natural logarithm (rank).

The mechanical ventilation database contained 14,629 disorder: intervention dyads. Incomplete pairs resulting from keystroke errors accounted for 0.6% and were excluded, leaving 14,552 dyads for analysis. We analyzed the last 25 virtual patients managed by each subject, comprising a database of 2926 dyads. Comparing the appearance frequency of each word to its rank order revealed a power law relationship. Plotting the natural logarithm of appearance frequency against the natural logarithm of rank order over approximately 6 orders of magnitude yielded a frequency: rank slope of -0.95 and an R^2^ of 0.96 (Figure 2).

### Dialysis

**Figure 3:**
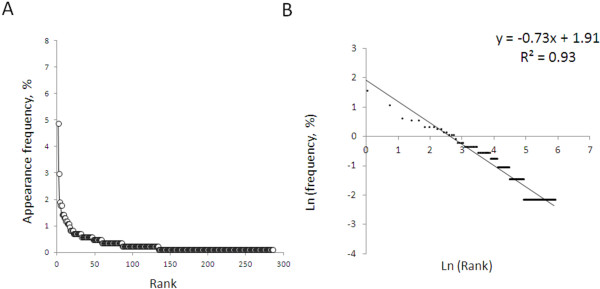
**Practice patterns during anemia management in end stage renal disease display language like statistical patterns.** Panel **A**: Word appearance frequency versus rank of word usage during anemia management in end stage renal disease. Panel **B**: natural logarithm (frequency) versus natural logarithm (rank) during anemia management.

797 unique exemplars of patient derangement: practitioner intervention were available for analysis. As above, combinatorial techniques were used to encode the observed pattern of patient derangement and the integrated pattern of the practitioner’s response. These symbols were then concatenated to form words, and the frequency of word usage in the population of practitioners was characterized. Comparing the appearance frequency of each word to its rank order revealed a power law relationship. Plotting the natural logarithm of appearance frequency against the natural logarithm of rank order over approximately 6 orders of magnitude yielded a frequency: rank slope of -0.73 and an R^2^ of 0.93 (Figure 3). An analysis at the individual level was not possible for this population, due to infeasibility of precisely ascribing interventions to unique providers.

### Power law behavior at the population level reflects similar behavior at the individual level

**Figure 4:**
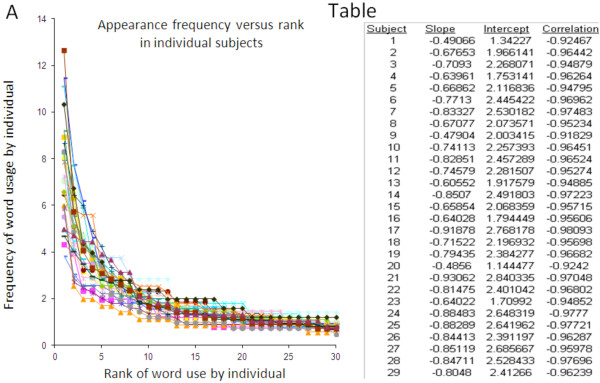
**Power law behaviour in word usage is observed at the individual level during simulated practice of mechanical ventilation.** Panel **A**: Word appearance frequency versus word appearance rank for each subject during simulated practice of mechanical ventilation. Table: Regression characteristics for natural logarithm (appearance frequency) versus natural logarithm (rank) for each subject.

An important question is whether the population level power law behavior reflected power law behavior at the individual level or was attributable to characteristics of the provider population and did not reflect power law behavior at the individual level. We used the mechanical ventilation data to address this issue, plotting overall word frequency versus word rank for each subject (Figure 4). These analyses revealed power law behavior at the level of individuals, with a mean slope of the natural logarithm of appearance frequency versus natural logarithm of rank of -0.74 (range, -0.48 to -0.93; standard deviation 0.13) and a mean correlation coefficient of -0.96 (range, -0.92 to -0.98, standard deviation 0.015).

### Observed intervention lengths did not fit either an intermittent silence or random text model

**Figure 5:**
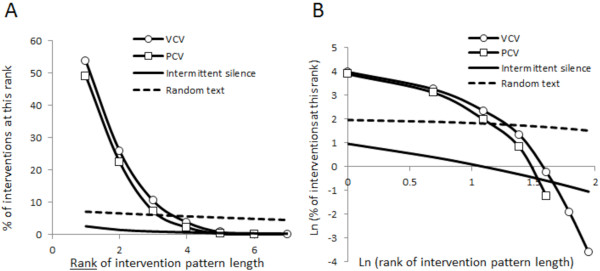
**Individual intervention pattern lengths do not follow the pattern predicted by intermittent silence or random text analyses.** Panel **A**: Appearance frequency of intervention pattern length versus rank for volume controlled and pressure controlled modes during simulated practice of mechanical ventilation and patterns predicted by intermittent silence and random text models. Panel **B**: natural logarithm (frequency) versus natural logarithm (rank) for observed data and those for the intermittent silence and random text models.

Plots of intervention length frequency versus intervention length rank for both volume controlled ventilation and pressure controlled ventilation were characterized by steep declines in frequency as rank number increased; this trajectory was smooth, did not follow a clear power law, and was much more marked than the behavior anticipated from implementing an intermittent silence model or a random text model (Figure 5).

### Observed solution lengths did not fit either an intermittent silence or random text model

**Figure 6:**
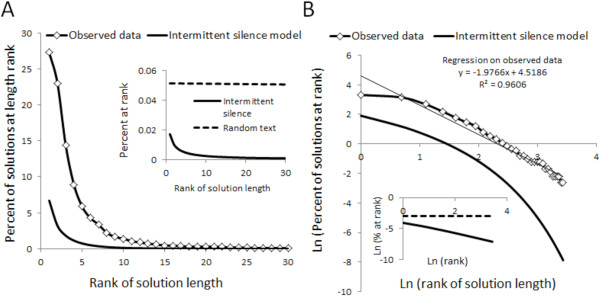
**Problem solution lengths do not follow the pattern predicted by intermittent silence or random text analyses.** Panel **A**: Solution length appearance frequency versus solution length appearance rank during simulated practice of mechanical ventilation and pattern predicted by intermittent silence model using observed space frequency. Inset: behavior predicted using intermittent silence model and space frequency of 5.1 × 10^-4^ and that predicted from random text model; note difference in scale. Panel **B**: natural logarithm (frequency) versus natural logarithm (rank) for observed data and that for the intermittent silence model using observed space frequency. Inset: behavior predicted using intermittent silence model and space frequency of 5.1 × 10^-4^ and that predicted from random text model.

The observed frequency of an effective space (patient completed) was 0.2. The observed data display a strong power law behavior that is much more dramatic than that anticipated in an intermittent silence model using 1946 keys and a pause frequency of 0.2 or a random text model (Figure 6). When an effective space frequency of 5.1 × 10^-4^ (corresponding to a purely random selection of the space bar) is used, the difference is more marked.

Our findings suggest that patterns of complex, multidimensional medical care can be captured using symbolic dynamic techniques, and that the quantitative languages thereby constructed display statistical similarities to natural languages. This appears to be a population level phenomenon reflective of behaviors at the individual level (Figure 4). Moreover, during the simulated practice of mechanical ventilation the “solution lengths” also followed a power law, and the observed trajectories of both intervention lengths and the solutions into which they are concatenated diverged sharply from those predicted by intermittent silence and random text models. The observation of power law behavior in the “languages” adopted by practitioners as they addressed problems in two vastly different domains of care and characterized by entirely different timescales suggests that this statistical finding may be germane to other arenas of titratable care.

Power law behavior has been observed in many settings, ranging from the growth of cities through income levels and including the “coding” of clinical diagnoses [20-23]. As noted in [18,19], there is debate regarding the extent to which such dynamics reflect meaningful dynamic underpinnings. In particular, Miller suggested that the power law behavior observed by Zipf was a consequence of “intermittent silences” punctuating a random process, and Li contends that the choice of rank as the independent variable accounts for the findings of Zipf.

In the case of languages, at least 3 mechanistic explanations for power law behavior have been put forth. In one, the natural tension between communicating parties leads to differential encoding between “sender” and “receiver,” with consequent power law behavior [24-27]. Alternatively, the principle of “selective attachment,” possibly acting through Pittman-Yor or similar mechanisms, may lead to clustering of “concepts” [28]. Nowack and Krakauer provide an evolutionary analysis of language development in which a limited number of phonemes, the potential for erroneous message transmission/interpretation, and the advantages of linking objects to actions leads to the development of languages having structure at multiple levels [29]. Finally, a combination of preferential attachment and “similarity” between sets of interventions may be at play [30].

Our finding that observed behaviors in the practice of mechanical ventilation deviate dramatically from those expected under either intermittent silence or random text analyses suggest that–in this context of problem solving–Zipf like behavior is not merely a consequence of intermittent silences or the selection of rank as the independent variable, but might reflect one of the alternative mechanisms enumerated above. Given that our data did not require “external communication,” we favor selective attachment mechanisms as the dominant factors in this context; however, the possibility that the practitioner is–to some extent–“speaking” to her/himself cannot be excluded, and would align with [27] and [29]. If this latter explanation holds, the multi-scale behaviors (“word length” and “solution length”) observed may share causes similar to the evolutionary pattern described by Nowak.

A data mining approach in which patient derangement: practitioner intervention dyads are encoded as symbol streams could prove useful for gauging the likely patient-specific efficacy of multicomponent therapeutic interventions (bedside decision support) as well as stratifying the potential effectiveness of multidimensional interventions at the population level. The power law behavior we observed suggests that, if data are continuously collected with periodic updating of the database, the rate of knowledge accrual will be greatest for more common problems, and will fall off in an exponential fashion as the frequency of specific derangement: intervention dyads declines.

In addition, the language-like statistical characteristics we observed may shed light on the cognitive structures underlying medical decision-making. Our subjects appear to have been “speaking mechanical ventilation” or “anemia management.” This suggests that characterizing the patterns of practice adopted by individual practitioners might be a natural approach to assessment of expertise or quality of care, an approach that could more effectively capture the nuances germane to management of individual patients. Similarly, the deviation of observed behaviors on a particular problem set from that anticipated in an intermittent silence model could provide a novel metric of the “difficulty” of the problems included in the set.

## Conclusions

Our results demonstrate that population level patterns of patient derangement:practitioner intervention word usage in two entirely unrelated domains of medical care display power law relationships identical to those of natural languages, and that- in one of these domains- power law behavior at the population level reflects power law behavior at the level of individual practitioners. In addition, the distribution of solution lengths in one of the domains does not follow that expected for a process driven by intermittent silence. Our results suggest that patterns of medical care can be approached using quantitative linguistic techniques, a finding that has implications for the assessment of expertise, machine learning identification of optimal practices, and construction of bedside decision support tools.

## Competing interests

The authors declared that they have no competing interests.

## Authors’ contributions

JRH, PSC, and JDP developed the mechanical ventilation simulator. JDP, CRB, and JRH developed the population of virtual patients. JDP and AMK recruited subjects for the ventilation study. JRH and PSC developed the mechanical ventilation and dialysis practice pattern characterization tools. JRH collected and collated the data for the dialysis study. JRH and PSC conducted the analyses. All authors read and approved the final manuscript.

## Pre-publication history

The pre-publication history for this paper can be accessed here:

http://www.biomedcentral.com/1472-6947/13/102/prepub

## Supplementary Material

Additional file 1 General algorithm for encoding. Click here for file
