# Supplementary material for: Medical practices display power law behaviors similar to spoken languages
Source: BMC Med Inform Decis Mak. 2013 Sep 4;13:102. doi: 10.1186/1472-6947-13-102 (PMC3766655; doi:10.1186/1472-6947-13-102)
Supplement: Additional file 1 — General algorithm for encoding. [file 1472-6947-13-102-S1.doc]

General algorithm for encoding

For each patient an acceptable range is defined. These may be dichotomous (for example, distending pressure within the lungs is acceptable or too high) or more finely graded (for example, arterial pH is too low, acceptable, or too high), and were drawn from common clinical practice. Controlled parameters were those the subject changed while attempting to attain specified physiologic goals, for example, changing the size of the breaths administered during mechanical ventilation. For analyses of practice patterns (Figures 2, 3, 4), the integrated physiology: provider response symbols were used; for examination of the individual utterances and solution lengths (Figures 5 and 6) the integrated response pattern symbols were used. Here is a simple 3-variable example of the encoding.

**1. A symbol is assigned to each physiologic variable at each point**

Value below goal range => symbol = “A”

Value within goal range => symbol = “B”

Value above goal range => symbol = “C”

**2. For each controlled parameter, a symbol is assigned to the change (if any)**

**implemented by the provider at that time point**

Parameter value increased => symbol =“W”

Parameter value not changed => symbol = “X”

Parameter value decreased => symbol = “Y”

**3. At each point, the symbols capturing the physiology are concatentated to form an integrated symbol, as are those describing the interventions. These can be combined to form a symbol describing the prevailing physiology and integrated provider response at that point**

Symbol for variable 1 Symbol for variable 2 Symbol for variable 3 Integrated physiology symbol

“A” “B” “C” “ABC”

Controlled parameter 1 Controlled parameter 2 Controlled parameter 3 Integrated response symbol

“W” “X” “Y” “WXY”

Integrated physiology: provider response symbol = “ABCWXY”

**4. Each time point is thus characterized by 3 different symbols: physiology, response, and integrated physiology: response**

Time point Time point 1 Time point 2 Time point 3

Physiology symbol ABC BBC BBB

Response symbol WXY XWX XXX

Integrated physiology: response ABCWXY BBCXWX BBBXXX
